# Cryopyrin-Associated Periodic Fever Syndrome and the Nervous System

**DOI:** 10.1007/s11940-018-0526-1

**Published:** 2018-09-03

**Authors:** Stephen Keddie, Thomas Parker, Helen J. Lachmann, Lionel Ginsberg

**Affiliations:** 1 Institute of Neurology, UCL, London, UK; 2 National Amyloidosis Centre, UCL, London, UK; 3 Department of Neurology, Royal Free Hospital, London, NW3 2QG UK

**Keywords:** CAPS, NLRP3, Aseptic meningitis, Canakinumab

## Abstract

**Purpose of review:**

The purpose of this review is to highlight the molecular and clinical characteristics of the cryopyrin-associated periodic fever syndrome (CAPS) and its management. CAPS is an autosomal dominantly inherited autoinflammatory disorder associated with mutations in the *NLRP3* gene, which ultimately lead to excessive production of interleukin-1β (IL-1β) and systemic inflammation. Typical systemic features include fever, urticarial rash and arthralgia, and ultimately amyloidosis. There are also multiple neurological manifestations including, but not restricted to, headache, sensorineural hearing loss, aseptic meningitis, myalgia and optic nerve involvement.

**Recent findings:**

Since the recognition of CAPS as a single disease entity and discovery of the underlying causative gene, there has been a major breakthrough in terms of its treatment by pharmacological IL-1β inhibition. Highly targeted therapies against IL-1 have been shown to be remarkably effective in the treatment of CAPS and make early diagnosis of this condition crucial. It is hoped that starting pharmacological intervention in a timely manner will prove neuroprotective. There are three drugs licensed for treatment of CAPS; canakinumab, anakinra and rilonacept. The former two are widely used: canakinumab is a fully humanised anti-IL-1β monoclonal antibody administered as a subcutaneous injection once every 8 weeks starting at a dose of 150 mg in patients weighing more than 40 kg. Anakinra is a recombinant form of the IL-1 receptor antagonist and the adult daily dose is 100 mg subcutaneously.

**Summary:**

CAPS is a highly debilitating disorder characterised by unregulated IL-1β production driven by autosomal dominantly inherited mutations in the *NLRP3* gene. Effective therapies targeted against IL-1 are now available and are vital to prevent long-term complications.

## Introduction

The cryopyrin-associated periodic fever syndrome (CAPS) is a multisystem genetic autoinflammatory disorder, which has a wide range of systemic as well as neurological manifestations. The cardinal symptoms of CAPS include episodes of fever, urticarial rash, red eyes and arthralgia [1], whilst neurological manifestations include headache, sensorineural hearing loss and optic nerve involvement [2••, 3, 4]. At the turn of the twenty-first century, the discovery of a common genetic locus, the *NLRP3* gene on chromosome 1q44, resulted in the recognition that a number of previously described clinical syndromes, including chronic infantile neurological, cutaneous and articular syndrome (CINCA), Muckle-Wells syndrome (MWS) and familial cold autoinflammatory syndrome (FCAS) were part of a wider clinical entity. The term CAPS began to gain recognition to describe the spectrum of autosomal dominant and apparently sporadic diseases caused by mutations in *NLRP3* [5, 6].

CAPS is rare and is estimated to affect one to three people per million of the population with no specific demographic predilection identified [7]. A large European registry [8••] has provided key clinical data regarding CAPS. Median age at onset was 0.8 years (interquartile range 0.1–5) with age at diagnosis of 15 years (IQR 5–36). Forty percent of patients experience a recurrent course, 40% chronic and 20% chronic with exacerbations. Most attacks resolve within 24 h (48%) but 36% can last more than 3 days. In terms of systemic features, skin involvement was the most commonly reported symptom (97%). Fever is seen in 84% and arthralgia in 86%. Patients still tend to be subdivided into FCAS, MWS and CINCA, although it is becoming increasingly clear that there is substantial overlap between these groups, and CAPS is best regarded as a continuous rather than discrete severity spectrum.

## Familial cold autoinflammatory syndrome

Symptoms consistent with the FCAS phenotype most commonly develop in the first few years of life and are typified by fever, urticaria, conjunctival inflammation and arthralgia, which can occur from 30 min to 48 h following cold exposure and resolve typically after a few hours. Secondary AA amyloidosis is very rare in FCAS, but despite being the mildest CAPS phenotype, FCAS often has a severe impact on patient quality of life with symptoms precluding outdoor activities in over 95% of patients [9].

## Muckle-Wells syndrome

MWS is now recognised to be an intermediate phenotype of CAPS characterised by chronic or intermittent episodes of fever, headache, urticarial like rash, red eyes, arthralgia or arthritis, often in the absence of a specific trigger [10]. Febrile attacks commence in early childhood and patients often develop progressive sensorineural hearing loss. Approximately 25% of untreated patients develop AA amyloidosis secondary to chronic inflammation [11].

## Chronic infantile neurological cutaneous and articular syndrome

CINCA is viewed as the most severe CAPS phenotype [6]. In general, children present during the first days of life with a chronic urticarial rash associated with persistent low-grade fever and elevation of acute phase reactants. Patients frequently have a typical facial appearance, with frontal bossing, large cephalic perimeter and saddle-back nose [12••]. They develop permanent neurological damage with chronic aseptic meningitis, raised intracranial pressure and hydrocephalus, brain atrophy and chronic papilloedema [8••]. Chronic irritability, intellectual disability, headache, lower limb spasticity and more rarely seizures can occur [10]. There can be eye inflammation from anterior to posterior including conjunctivitis, uveitis and optic neuritis. Papilloedema due to raised intracranial pressure and optic neuritis can result in optic atrophy [13]. Cochlear inflammation leads to progressive stepwise sensorineural hearing loss in the first years of life. Approximately 60% of patients have prominent arthropathy, usually during childhood, causing deformity and degenerative arthropathy leading to contractures [12••]. Mortality is high, and death in childhood well recognised. Chronic inflammation leads to the deposition of AA amyloid and renal failure [12••].

## Molecular pathogenesis of CAPS

**Fig. 1:**
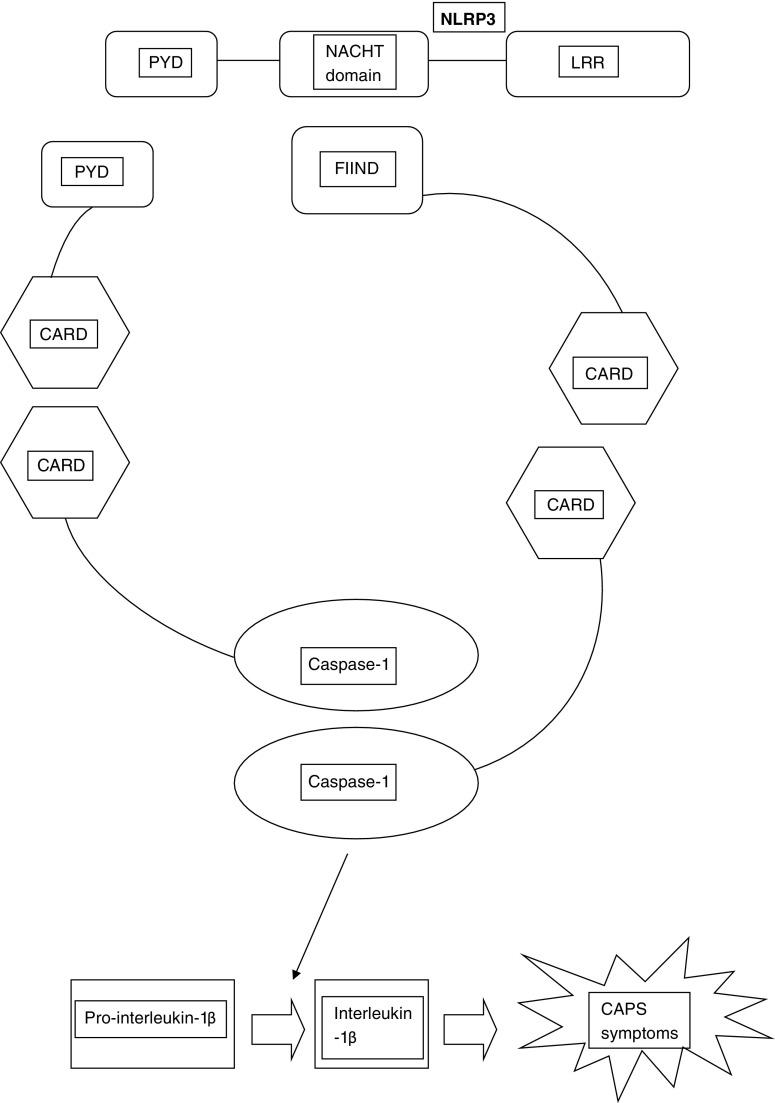
Schematic representation of the *NLRP3* inflammasome. The activation of *NLRP3* by a variety of pathogen-associated molecular pattern molecules interacting with the leucine-rich repeat (LRR) domain leads to aggregation and oligomerisation of a molecular complex which in turn activates caspase-1, thereby promoting the production of the proinflammatory cytokine interleukin-1β. In CAPS, gain-of-function mutations of *NLRP3*, often in the NACHT domain, result in overactivation of this pathway. Other domains of the assembling macromolecule labelled in the diagram are CARD (caspase activation and recruitment domain), PYD (pyrin domain) and FIIND (“function-to-find” domain).

CAPS is caused by heterozygous gain-of-function mutations within the *NLRP3* gene, located on chromosome 1q44, which can be inherited in a dominant fashion with variable penetrance and expression, or arise de novo, the latter usually in CINCA. *NLRP3* encodes cryopyrin, a member of the intracellular nucleotide-binding oligomerisation domain-like receptors (NLRs). NLRs are pattern recognition receptors which recognise a variety of pathogen-associated molecular pattern molecules (PAMPs) [10]. All NLRs contain a NACHT domain (common to **NA**IP (neuronal apoptosis inhibitory protein), **C**IITA (MHC class II transcription activator), **H**ET-E (incompatibility locus protein from *Podospora anserina*) and **T**P1 (telomerase-associated protein)) that regulates autoaggregation and oligomerisation. Oligomerisation of cryopyrin results in activation of caspase-1. This catalyses the cleavage of prointerleukin-1β to interleukin-1β, and is the key step in its regulation. Interleukin-1β is a potent proinflammatory cytokine with pleiotropic effects including fever, vasodilatation and production/activation of other inflammatory cytokines [7]. Mutations in *NLRP3* therefore cause overactivation of the IL-1β pathway, a cascade of complex cellular events and ultimately aberrant homeostatic tissue responses (Fig. 1) [8••, 10].

Around 170 *NLRP3* variants have been associated with CAPS. The majority of mutations identified have been observed within exon 3, which is responsible for encoding the NACHT domain, crucial for cryopyrin oligomerisation [12••]. In CINCA, mutations in other exons encoding the leucine rich repeat PAMP recognition domain are also known. Although patients with FCAS and MWS tend to show familial inheritance, CINCA usually occurs de novo. Approximately 50–65% lack mutations in *NLRP3* by conventional Sanger sequencing. It is now recognised that many of these are mosaics for very damaging mutations, but other genes are also likely to be implicated in the phenotype [12••].

## Neurological manifestations of CAPS

The variety of neurological manifestations reported in the literature highlights the importance of considering CAPS as a potential differential diagnosis in a wide range of neurological presentations.

A selection of articles that report the neurological manifestations of CAPS is summarised in Table 1. Key neurological features of CAPS include headache, with a notably high proportion of patients with phenotypic features consistent with migraine or chronic daily headache [2••, 3, 4]. In addition, more sinister headache phenotypes with evidence of raised intracranial pressure and aseptic meningitis have been reported [2••, 4, 8••]. Papilloedema is seen in around 30% of patients [2••, 3, 8••].

**Table 1:** Examples of papers describing neurological manifestations of CAPS (order of year of publication)

Citation	Study type	Key findings
[4]	Patient cohort national referral centre (UK) (*n* = 13)	• Headache present in 92% (77% had features of migraine) • Sensorineural deafness present in 54% • Myalgia reported in 69% • 46% had papilloedema and 15% had optic disc pallor
[8••]	Web-based Eurofever Registry (*n* = 136)	• 40% classified as having neurological features (morning headache, papilloedema and aseptic meningitis to seizures and hydrocephalus) • Ophthalmological involvement in 71% and sensorineural hearing loss in 42%.
[14]	Patient cohort (*n* = 17)—screening of 108 patients in neuroimmunology clinic (Germany)	• 65% had severe headache syndromes (55% were diagnosed with migraine) • 53% had a concomitant diagnosis of multiple sclerosis • 56% of those receiving treatment responded to anti-interleukin-1 therapy.
[15]	Single case report	• Chorea associated with chronic white matter lesions on FLAIR imaging with partial gadolinium enhancement • Good response to anakinra
[2••]	Patient cohort—national referral centre (UK) (*n* = 38) (expanded cohort from ref. [4])	• 84% had some form of headache • 66% sensorineural hearing loss • 60% myalgia • 34% papilloedema and 26% optic atrophy • 90% of patients with headache responded to IL-1 inhibition
[16]	Single case report	• Unilateral orbital pain and diplopia with granulomatous inflammation of both cavernous sinuses on brain MRI consistent with Tolosa-Hunt syndrome • CSF pleocytosis noted after lumbar puncture • Only partial response to prednisolone • Genetic testing revealed *NLRP3* mutation
[3]	Patient cohort—national referral centre (France) (*n* = 24)	• 17 patients (71%) had neurological involvement (predominantly headaches and hearing loss) • 11 adult patients (61%) and 3 children (50%) had school difficulties

Sensorineural hearing loss occurs in approximately 40% of patients, many requiring hearing aids or cochlear implantation. As hearing loss onset is often in mid to late childhood, many of these patients acquire normal speech. Mild ocular manifestations such as conjunctivitis (66%) or uveitis (7%) are frequently reported [8••]. Severe ocular involvement, including optic atrophy, cataract and impaired vision, is less common [2••, 3, 4, 8••].

Although the numbers of patients are small, there is evidence that specific genotypes may influence the extent to which neurological symptoms manifest. Data from the cohort based in the UK suggested patients with the T348M mutation tended to have a more severe neurological phenotype with an earlier age of onset [2••]. Data from an observational study in France were consistent with the UK data and found that the R260W mutation was more likely to be associated with neurological involvement in patients compared to those with the A439V mutation [3]. The E311K mutation is reported to be associated with a higher rate of hearing loss [17].

In one interesting study, 108 patients in a neuroimmunology clinic were screened for *NLRP3* mutations on the basis of systemic symptoms compatible with mild CAPS. The authors reported 17 (16%) patients had a V198M or Q703K *NLRP3* variant [14]. The interpretation of these variants is contentious as they occur at a high frequency in the healthy population; V198M has an allele frequency of 0.007, and Q703K is seen in 10% of healthy controls. In vitro functional studies have suggested that V198M has an intermediate effect on inflammasome activation and IL-1β production compared to definite pathogenic mutations and wild-type controls. Many of these patients reported neurological features (e.g. headache and sensorineural hearing loss). Nine patients underwent anti-IL-1 therapy, with 5 reported to have responded to treatment [14].

A single case report recently described the diagnosis of CAPS in a patient with Tolosa-Hunt syndrome after genetic testing [16], whilst another case report highlighted chorea as a manifestation of CAPS [15]. Cognitive dysfunction may also be a feature of CAPS, with a study from a specialist centre in France reporting educational difficulties in the majority of adults (61%) and children (50%) [3].

## Management of CAPS

A suggested approach to the management of CAPS is shown in Table 2. Of prime importance once a diagnosis is genetically confirmed is the institution of disease modifying therapy in the form of IL-1 inhibition. Canakinumab is a fully human anti-IL-1β monoclonal antibody administered as a subcutaneous injection once every 8 weeks starting at a dose of 150 mg. In a double-blind placebo-controlled randomised withdrawal study, there was evidence of a complete response to canakinumab in 78% of drug-naive patients [18, 19]. Monitoring of systemic disease activity in the form of serum amyloid-A protein (SAA) levels is the most sensitive marker of CAPS disease activity and predictor of the risk of systemic AA amyloidosis. Ninety percent of patients treated with canakinumab showed complete normalisation of SAA by day 8 post treatment, as well as concomitant decrease in C-reactive protein (CRP) and resolution of symptoms [18, 19].

**Table 2:** Key management recommendations for cryopyrin associated periodic fever syndrome (CAPS)

Recommendation	Details
Referral to specialist centre	• UK: The National Amyloidosis Centre, Royal Free Hospital, London http://www.ucl.ac.uk/amyloidosis/nac
Genetic analysis	• Genetic counselling • Testing of *NLRP3* gene • Consideration of family member screening
Lifelong IL-1 inhibition	• First line: subcutaneous injection of canakinumab every 8 weeks (starting dose 150 mg) • Second line: subcutaneous injection of anakinra once daily (starting dose 100 mg)
Regular monitoring of disease activity	• Quality of life questionnaire • Serum amyloid A (SAA) measurement • Serum C-reactive protein (CRP) measurement
Monitor for complications	• Baseline neurological assessment with brain MRI (repeat brain MRI in those with abnormalities and consideration of lumbar puncture depending on clinical presentation) • Annual pure tone audiometry • Annual ophthalmic assessment by ophthalmologist • Serum amyloid P (SAP) scan if suggestion of systemic amyloidosis
Patient education	• Clinical nurse specialists • Online communities e.g. https://www.rareconnect.org/en/community/caps

In addition to canakinumab, anakinra, a recombinant form of the IL-1 receptor antagonist, delivered as a daily dose of 100 mg via subcutaneous injection [20], is also licensed in CAPS. Anakinra was the first commercially available anti-IL-1 agent and has been shown to be effective against IL-1-driven autoinflammatory disease [20, 21].

CAPS is a lifelong illness that affects women of child bearing age, and optimal control of symptoms during pregnancy is of utmost importance for the health of both mother and foetus. There are safety data available for anakinra in pregnancy [22]. An observational study of 15 pregnancies reported no preterm births or serious complications of pregnancy using anakinra throughout gestation, whilst providing relief of CAPS symptoms. An international collaborative study has reported that paternal exposure to IL-1 blockade appears uncomplicated in an experience of 12 cases to date. Maternal data on 23 women exposed to anakinra and 8 exposed to canakinumab are generally reassuring but the numbers remain small and there is concern about renal agenesis [23••]. Experience is that many patients are unable to discontinue treatment due to rapid recurrence of autoinflammatory disease, and individual preconception risk benefit discussion with prospective parents is strongly recommended.

As previously highlighted, headaches, and in particular migraine, are a common neurological manifestation of CAPS [2••, 3, 4]. Migraines are also common in the wider population [24] and may be unrelated to underlying CAPS pathology. It is therefore important to correlate headache and other migrainous symptoms with systemic disease activity, and in many cases migraines may resolve with disease modification [2••]. However, effective symptomatic and preventative treatments for migraine and other primary headache disorders are available and should be considered in addition to disease modification in CAPS on a patient by patient basis, using international guidelines [25].

Hearing loss is a common manifestation of CAPS. A recent study where CAPS patients underwent detailed auditory testing found that 100% of symptomatic CAPS patients had high frequency pure tone averages abnormalities, whilst 74% had an abnormal assessment focusing on standard frequencies (0.5–4 kHz) [26]. Encouragingly, treatment with IL-1 blockade resulted in improved or stable hearing in 91% of patients [26]. However, it is important to note that once sensorineural damage has occurred, hearing dysfunction is likely irreversible. This highlights the importance of regular and detailed auditory screening, as well as early institution of disease modification to prevent CAPS-related hearing loss. Beyond prevention, advances in hearing aids and cochlear implants are ongoing [27], and obtaining the relevant opinions from otolaryngology and audiology colleagues is of paramount importance in patients with established hearing loss. Similarly, input from ophthalmology colleagues is crucial as eye involvement at any level from the cornea to the optic nerve is common and can be sight threatening [2••, 4, 8••]. We would recommend annual assessment in all cases, with more frequent assessment if abnormalities are detected.

Although the majority of CAPS patients have clinically normal brain MRI [2••, 4], cranial imaging can be helpful as white matter hyperintensities are sometimes observed [2••, 3, 14, 15], and in some patients, have been reported to improve after anti-IL-1 treatment [15]. Interestingly, in the aforementioned cohort of patients from a neuroimmunology clinic, a much higher proportion of patients (88%) had MRI brain abnormalities, often in the form of white matter lesions [14].

In addition to the neurological manifestations, monitoring of systemic symptoms is important. If there is a clinical suspicion of systemic AA amyloidosis, which can be ultimately life threatening in the form of end-stage renal failure [11], a renal biopsy or serum amyloid P (SAP) nuclear medicine scan is advised [28].

Management of CAPS is therefore complex and requires significant expertise and multidisciplinary involvement. In the UK, a national service is provided by the NHS National Amyloidosis Centre based in London (http://www.ucl.ac.uk/amyloidosis/nac) and referral to such centres is key to ensuring optimal management.

## Conclusions

Much is known about CAPS at a molecular and genetic level, but due to the non-specific, chronic, relapsing-remitting nature of the condition, patients’ diagnosis and treatment are often delayed. Once thought to be three separate entities, CAPS is now considered a continuous clinical spectrum of a single condition, with genotype-phenotype variations. Recurrent flares of fever, skin and joint involvement and neurological symptoms should lead a clinician to consider the diagnosis of CAPS, particularly in those with a positive family history. Neurological manifestations range from the most commonly occurring headache and sensorineural hearing impairment to severe features such as hydrocephalus and aseptic meningitis. Systemic inflammatory markers are elevated during exacerbations, as are levels of serum amyloid A protein. MR imaging may show non-specific white matter abnormalities. Diagnostic criteria with a sensitivity of 81% and specificity of 94% have recently been proposed [29]. These include raised inflammatory markers (CRP/SAA) plus at least two of six CAPS-typical symptoms (urticaria-like rash, cold-triggered episodes, sensorineural hearing loss, musculoskeletal symptoms, chronic aseptic meningitis and skeletal abnormalities) [29]. Diagnosis is confirmed by genetic testing for the *NRLP3* mutation. The fully human anti-IL-1β monoclonal antibody canakinumab or the recombinant form of the IL-1 receptor antagonist anakinra are highly effective medications preventing further episodes of systemic inflammation and transforming quality of life, but will not reverse established damage such as hearing or visual impairment. Symptomatic treatment of pain, headaches and skin rash should also be provided. Identification of affected patients early in the course of the disease is crucial to prevent irreversible damage or the development of AA amyloidosis.
